# Metabolic reprograming of LPS-stimulated human lung macrophages involves tryptophan metabolism and the aspartate-arginosuccinate shunt

**DOI:** 10.1371/journal.pone.0230813

**Published:** 2020-04-08

**Authors:** Fanta Fall, Elodie Lamy, Marion Brollo, Emmanuel Naline, Natacha Lenuzza, Etienne Thévenot, Philippe Devillier, Stanislas Grassin-Delyle

**Affiliations:** 1 Infection et inflammation, Université Paris-Saclay, UVSQ, INSERM, Montigny le Bretonneux, France; 2 Laboratoire Mécanismes moléculaires et pharmacologiques de l’obstruction bronchique, Université Paris-Saclay, UVSQ, Suresnes, France; 3 Hôpital Foch, Département des maladies des voies respiratoires, Suresnes, France; 4 Laboratory for Data Sciences and Decision, CEA, LIST, MetaboHUB, Gif-sur-Yvette, France; University of Michigan Health System, UNITED STATES

## Abstract

Lung macrophages (LM) are in the first line of defense against inhaled pathogens and can undergo phenotypic polarization to the proinflammatory M1 after stimulation with Toll-like receptor agonists. The objective of the present work was to characterize the metabolic alterations occurring during the experimental M1 LM polarization. Human LM were obtained from resected lungs and cultured for 24 hrs in medium alone or with 10 ng.mL^-1^ lipopolysaccharide. Cells and culture supernatants were subjected to extraction for metabolomic analysis with high-resolution LC-MS (HILIC and reverse phase -RP- chromatography in both negative and positive ionization modes) and GC-MS. The data were analyzed with R and the Worklow4Metabolomics and MetaboAnalyst online infrastructures. A total of 8,741 and 4,356 features were detected in the intracellular and extracellular content, respectively, after the filtering steps. Pathway analysis showed involvement of arachidonic acid metabolism, tryptophan metabolism and Krebs cycle in the response of LM to LPS, which was confirmed by the specific quantitation of selected compounds. This refined analysis highlighted a regulation of the kynurenin pathway as well as the serotonin biosynthesis pathway, and an involvement of aspartate-arginosuccinate shunt in the malate production. Macrophages M1 polarization is accompanied by changes in the cell metabolome, with the differential expression of metabolites involved in the promotion and regulation of inflammation and antimicrobial activity. The analysis of this macrophage immunometabolome may be of interest for the understanding of the pathophysiology of lung inflammatory disesases.

## Introduction

Lung macrophages are in the first line of defense against inhaled pathogens. They are major effectors of the immune response as they express membrane receptors, including Toll-like receptors (TLRs), able to recognize conserved microbial ligands [1]. Once activated, TLRs induce the production of a pattern of cytokines, chemokines and mediators, such as metabolites from the arachidonic acid pathway, involved in the inflammatory response and characteristics of a macrophage engagement towards a M1 polarization state [1, 2]. Lipopolysacharide (LPS) is the archetypal TLR ligand for the induction of M1 macrophage polarization [1]. Like allergens (e.g. ragweed pollen, house dust extract, and cat dander) or air pollutants, LPS binds to and activates TLR4, the first subtype among the TLR family to be identified in humans. Beyond this role in cell signaling, TLRs also play a role in the primary function of macrophages, consisting in the phagocytosis and killing of pathogens [3]. For phagocytosis, the production of hydrolytic enzymes and reactive oxygen species (ROS) is required, the latter also resulting from oxidase or lipoxygenase enzyme metabolism [4]. The enzymatic machinery is therefore modulated following the presence of microorganisms, and more generally by the external environment, and consequently the resulting production of metabolites varies, being likely to adapt to the different stimuli to which macrophages can be exposed [5]. Cell metabolism and macrophages functions are tightly linked, as already shown in murine bone-marrow-derived macrophages. Hence, the presence of PGE_2_, one on the main arachidonic acid derivative, is necessary to the LPS-induced production of the precursor of the pro-inflammatory cytokine IL-1β [6]; LPS also induces an increased glycolysis and succinate production, necessary to the increase of IL-1β expression and also driving the production of ROS [7, 8]; finally, the TLR4 agonist MPLA induces mouse resistance to systemic infection with *Staphylococcus aureus* and *Candida albicans* by reprogramming macrophage metabolism, with increased glycolysis and oxidative phosphorylation and rewiring of malate/NADH shuttling [9]. Therefore, the understanding of macrophage metabolic reprogramming has become a key focus in fields such as infection [10], inflammation [11], cancer [12] and immune disorders [13]. With respect to lung pathogenesis, macrophages also play a role in infections and inflammatory diseases such as asthma and chronic obstructive pulmonary disease (COPD), where they can undergo a phenotypic differentiation [14–17]. In these cases, TLRs are of prime importance for their role in the recognition of pathogens during infections and in microbe-induced acute exacerbations of asthma and COPD [18, 19]. However, the only reports of macrophage metabolic reprograming in lung diseases until now were in a mouse model of fibrosis [20], in alveolar macrophages from LPS treated mice [21] and in smoker’s or/and *Mycobacterium tuberculosis*-infected alveolar macrophages [22]. Since metabolic changes associated with the stimulation of TLRs in human lung macrophages were not yet described, the objective of the present study was to perform an extensive intra- and extracellular metabolomic characterization of LPS-induced alterations in human lung macrophages using a combined untargeted liquid chromatography high-resolution mass spectrometry and gas chromatography mass spectrometry approach [23].

## Materials and methods

### Patient population

The use of resected lung tissue was approved by the regional investigational review board (Comité de Protection des Personnes Ile de France VIII, Boulogne-Billancourt, France) and the patients undergoing surgical lung resection gave their written informed consent. Lung tissue was obtained from 10 patients with the following demographic characteristics: (median [25^th^-75^th^ percentiles]) age: 67.5 years [56.75–75.5]; 7 males, 3 females; body mass index: 24 kg/m^2^ [18.75–26.25]; current tobacco smokers/ex-tobacco smokers/pipe smoker: 4/6/1; pack-years: 30 [15–80]; and % FEV1 predicted: 109% [81–121]. One patient was suffering from COPD (as defined by a post-bronchodilator FEV1/FVC ratio <0.7; GOLD 2 stage) and none had undergone chemotherapy or radiotherapy prior to surgical lung resection.

### Reagents

Penicillin, streptomycin, L-glutamine, LPS from *Escherichia coli* (serotype K12), fatty acid methyl esters (FAMEs), LC-MS-grade ammonium formate and formic acid (98%) were purchased from Sigma Aldrich (Saint Quentin Fallavier, France). RPMI 1640 medium, phosphate-buffered saline, and fetal calf serum (FCS) were obtained from Eurobio Biotechnology (Les Ulis, France). LC-MS-grade methanol, acetonitrile, chloroform, isopropanol and water were from Fisher Scientific (Illkirch, France). All cell culture plastics were purchased from CML (Nemours, France).

### Cell culture

Human lung macrophages were isolated and cultured as previously described [1]. 2 million cells were cultured for 24 hrs in medium alone or with 10 ng.mL^-1^ LPS. Supernatants were collected and centrifuged at 2000 rpm for 5 min at 4°C, then frozen at -80°C. Adherent macrophages were washed twice with sterile cold PBS. For LC-MS analysis, the macrophages were collected with 500 μL of methanol/water (1:1); for GC-MS analysis 1 mL of acetonitrile/isopropanol/water (3:3:2) was used. The plates were left at -80°C for 20 min then scraped with a pipette tip to recover the cells and samples were kept at -80°C until analysis.

### Metabolomic analysis

#### Liquid chromatography-high resolution mass spectrometry

Sample preparation was adapted from the method described by Bligh and Dyer [24]. For supernatants, 500 μL of methanol/water (1:1) were added to 100 μL of culture medium. Then, for both supernatants and cells, 500 μL of chloroform were added. The mixture was sonicated for 10 min, stir for 10 min and centrifuged at 10000 rpm for 5 mins to achieve a biphasic separation with the upper phase containing polar compounds and the lower fraction nonpolar compounds. 2x200 μL of each phase were collected in Eppendorf tubes and dried under vacuum. One of each 4 dried extracts was reconstituted with 75 μL of each of the following mixtures: formate/acetonitrile (20:80 *v/v*) and carbonate/acetonitrile (20:80 *v/v*) for extracts from the upper phase subsequently analysed with hydrophilic interaction liquid chromatography (HILIC); formate/acetonitrile (80:20 *v/v*) and carbonate/acetonitrile (80:20 *v/v*) for extracts from the lower phase subsequently analysed with reverse phase (RP) chromatography. Quality control samples were prepared by pooling 5 μL of each extracted sample.

LC-HRMS analysis was adapted from a previously described method [23] and each sample was injected four times, with HILIC and RP chromatrography, both in the negative and positive ionization modes. Chromatography was performed with an UltiMate 3000 Quaternary Rapid Separation Pump (Thermo Scientific Dionex, Les Ulis, France) and the separation was performed under gradient elution using 4 different mobile phase systems consisting of mixtures of acetonitrile with either solvent A (10 mM pH 3.8 ammonium formate) for the positive ionization mode or solvent B (20 mM pH 9.2 ammonium carbonate) for th e negative ionization mode.

A SeQuant 4.6 mm x 150 mm, 5 μm i.d. ZIC-pHILIC column (AIT France, Houilles, France) was used for HILIC chromatography. For the positive ionization mode, gradient started with 5% solvent A until 3 min, then increased to reach 95% at 25 min and maintained for 5 more mins, then back to 5% and equilibrated for 10 mins. For the negative ionization mode, gradient started with 8% solvent B until 3 min, then increased to reach 92% at 25 min and maintained for 5 more mins, then back to 8% and equilibrated for 10 mins. Flow rate was 0.3 mL/min, curve gradient parameter set at 5, oven temperature 40°C and total run time 40 mins.

A Hypersil Gold C18 column 2.1 mm x 100 mm, 1.9 μm i.d. (Thermo Scientific Dionex) was used for RP chromatography. For the positive ionization mode, gradient started with 90% solvent A until 3 min, then decreased to reach 5% at 25 min and maintained for 8 more mins, then returning to 90% A for 5 mins. For the negative ionization mode, gradient started with 90% solvent B until 3 min, then decreased to reach 5% at 25 min and maintained for 8 more mins, then returning to 90% B for 5 mins. Flow rate was 0.3 mL/min, oven temperature 40°C and total run time 40 mins.

Mass spectrometry was performed with an hybrid quadrupole–orbitrap Q-Exactive mass spectrometer (Thermofisher) equipped with an heated electrospray ionization (ESI) source operating in positive (ESI+) or negative (ESI-) ionization modes. The ESI and acquisition parameters for the different modes are shown in S1 Table. Auxiliary gas heater temperature was set at 100°C, resolution at 70,000 and AGC target at 10^6^. The acquisition scan-range was split into 3 segments as previously described [23]: *m/z* 60–300; 300–600; 600–900. Xcalibur software (Thermofisher) was used for system controlling and data acquisition.

#### Gas chromatography-mass spectrometry

Samples were prepared according to the method described by Fiehn [25]. For supernatants, 1 mL of acetonitrile/isopropanol/water (3:3:2) was added to 30 μL of medium. The supernant and cell samples were then vortexed for 10 s, shaken for 5 mins, centrifuged at 14 000 rpm for 2 mins. 450 μL were recovered and dried under vacuum. The residue was reconstituted with 450 μL of acetonitrile/water 50:50 (1:1) degassed with nitrogen and centrifuged for 2 mins at 14 000 rpm. The residue was reconstituted with 10 μL of methoxyamine (MEOX) (20 mg/mL in pyridine), vortexed for 30 s, and kept at 30°C for 90 mins. Finally, 75 μL of N-methyl-N-trimethylsilyl-trifluoroacetamide (MSTFA) spiked with a mixture of FAME internal standards (10 μL of FAMEs for 1 mL of MSTFA) were added and the extract kept at 37°C for 30 mins. After derivation the samples were immediately transferred into injection vials.

Gas chromatography was performed on a Trace 1300 system (Thermofisher) with an Uptibond 5 Premium column 30 m x 0.25 mm x 0.25 mm (Interchim, Montluçon, France) and helium as a carrier gas at a flow rate of 1 mL/min. The injection volume was 1 μL. The temperature ramp was as follows: 60°C for 1 min, then an increase up to 325°C at 10°C/min. This temperature was maintained for 10 mins and total acquisition time was 37.5 mins. The detection was performed with a TSQ8000 mass spectrometer (Thermofisher). The electron impact (EI) ion source was held at 230°C and an energy of 70 eV was used. Acquisition was performed in the full scan mode (*m/z* 50–600) with an acquisition rate of 20 spectrum/s. Xcalibur software (Thermofisher) was used for system controlling and data acquisition.

#### Data processing

LC-MS raw files were first converted to mzML and centroidized with msConvert [26], then processed using IPO [27] and XCMS (v1.50.1) [28] packages running under R. The CentWave algorithm [29] was used for automatic peak picking, with parameters optimized with IPO. For GC-MS, the raw files were converted to CDF format. The data were analyzed with Workflow4Metabolomics using the metaMS, XCMS and CAMERA packages [28, 30, 31] that extract the peaks, align them, correct the analytical drift and perform annotation of adducts and isotopes.

Features detected in biological samples with a mean intensity less than 3-fold the intensity observed in blank samples, or features detected in blank samples only were filtered out to limit the number of false positive peaks. Features with a CV greater than 30% in the quality control samples were also filtered out. Batch correction, quality control checks and statistics were performed with Workflow4Metabolomics [32, 33]. Statistical analysis was also performed with Workflow4Metabolomics and MetaboAnalyst 4.0 using uni- or multivariate analysis and pathway analysis based on Mummichog [34]. Levels of metabolite annotation were defined as follows: level 0, which is the strongest level of annotation and includes stereochemistry discrimination; level 1 that requires the use of a chemical standard and at least two orthogonal techniques (e.g., accurate mass and retention time); level 2 is confirmation by a class-specific standard; level 3 by one parameter (e.g., accurate mass); level 4 is the feature-level without annotation [35]. HMDB, The Golm Metabolome Database and NIST [36, 37] were used for database queries.

## Results

### Untargeted metabolomic analysis

For the intracellular metabolome, the combination of the different LC-MS and GC-MS methods allowed the detection of 42,573 features in control and LPS-treated samples (Table 1). HILIC was the most contributive, followed with RP chromatography and GC-MS and a total of 8,741 features were remaining after the filtering steps.

**Table 1:** Features detected in the intracellular metabolome analysis.

Analysis	Chromatography	Polarity	Initial number of features	After Filtering
LC-MS	HILIC	+	16,588	2,935
		-	11,613	4,592
	RP	+	9,772	867
		-	4,407	154
GC-MS	GC	NA	-	193

For the extracellular metabolome, 45,258 features were first detected, with 4,356 remaining after filtering (Table 2). RP chromatography allowed the detection of the highest number of features, followed with HILIC and GC-MS.

**Table 2:** Features detected in the extracellular metabolome analysis.

Analysis	Chromatography	Polarity	Initial number of features	After Filtering
LC-MS	HILIC	+	11,411	840
		-	17,821	1,055
	RP	+	14,426	2,160
		-	1,326	27
GC-MS	GC	NA	-	274

### Multivariate analysis

**Fig 1:**
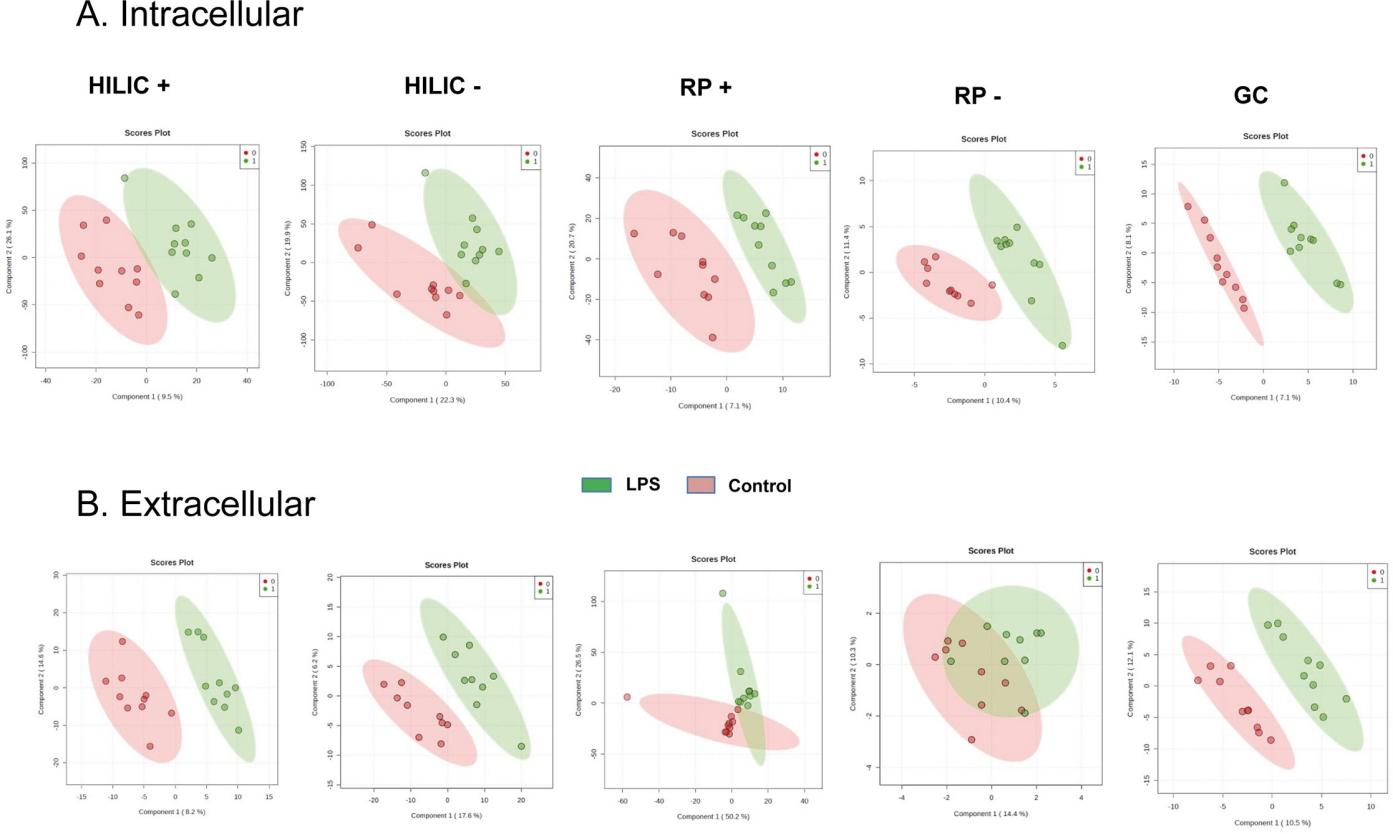
Partial Least Squares—Discriminant Analysis (PLS-DA). PLS-DA was performed for the intracellular (A) and extracellular (B) HILIC, RP and GC-MS analysis of the human macrophage metabolome (*n* = 10) in cells cultured for 24 hrs with (green) or without (red) LPS (10 ng.mL^-1^). HILIC: hydrophilic interaction liquid chromatography; RP: reverse phase; GC: gas chromatography.

**Fig 2:**
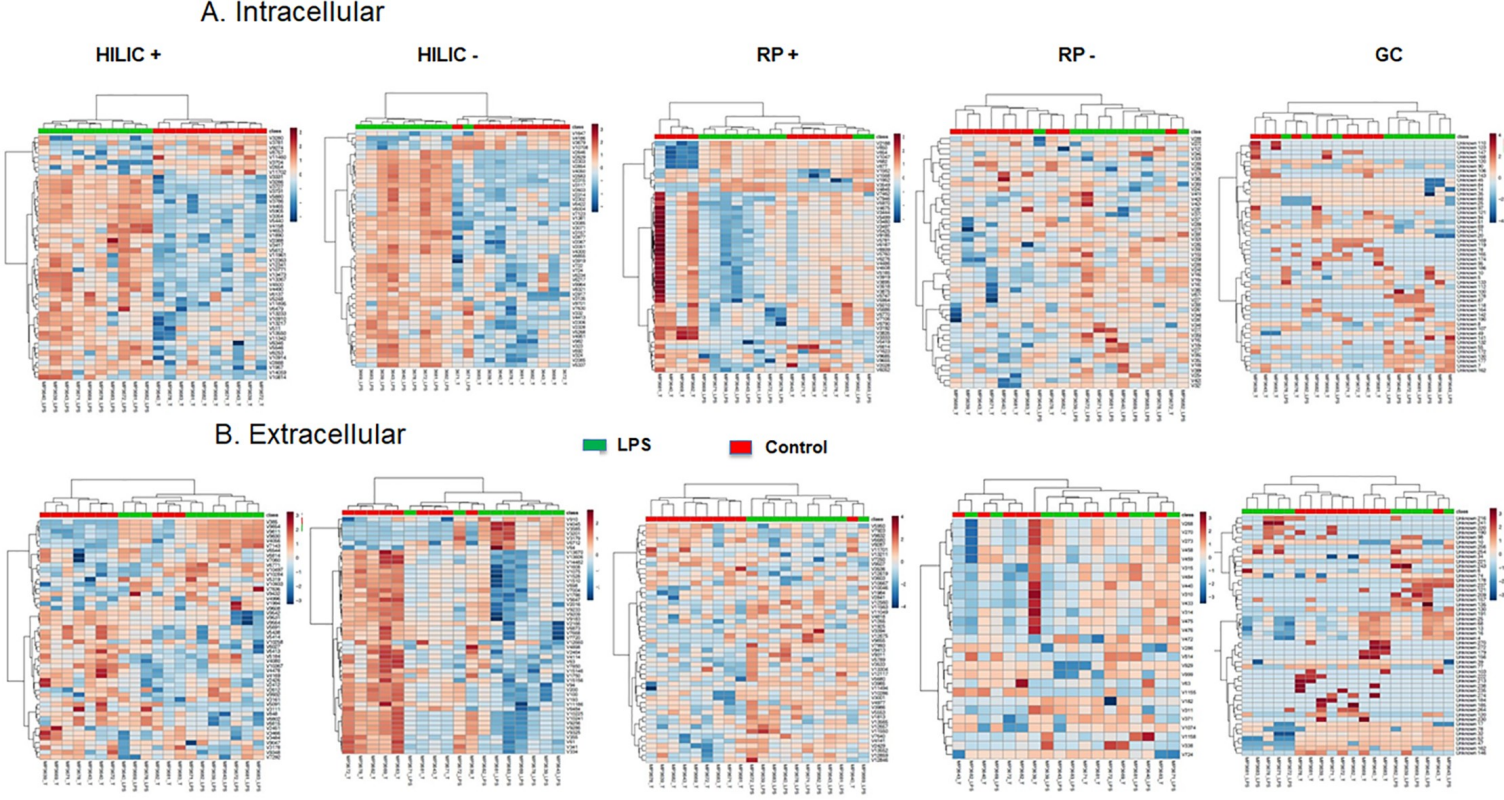
Hierarchical clustering analysis. The top 50 differentially expressed metabolites for the intracellular (A) and extracellular (B) HILIC, RP and GC-MS analysis of the human macrophage metabolome (*n* = 10) in cells cultured for 24 hrs with (green) or without (red) LPS (10 ng.mL^-1^) were used for hierarchical clustering. Metabolites in red are upregulated, those in blue downregulated.

Models were built with Partial Least-Squares—Discriminant Analysis (PLS-DA) for each condition and are shown in Fig 1A and 1B for the intracellular and extracellular metabolomes, respectively. The models for the intracellular metabolome allowed the distinction between control and treated cells with taking into account 2 components for the four analytical conditions. Each of these 2 components explained between 7.1 and 26.1% of the variability. On the other hand, with respect to the extracellular metabolome, 2 components were enough to discriminate control and treated cells for HILIC in the positive ionization mode, but 3 components were suggested by cross-validation to discriminate samples categories in the other analytical conditions. Although all models well fitted the data (R2Y > 0.9), their prediction capacity was poor with Q2Y < 0.3, except for the analysis of the extracellular content with HILIC in the positive ionization mode (Q2Y = 0.5). The features contributing the most to each model (assessed with Variable Importance in Projection (VIP) score) were then used for hierarchical clustering and for pathway analysis. Hierarchical clustering for the intracellular and extracellular metabolomes is depicted in Fig 2A and 2B, showing that an excellent categorization was achieved between control and LPS-treated macrophages for the majority of the different analytical conditions. Two categories of features, i.e. up- or down-regulated in control or LPS groups, are clearly distinguishable for the analysis of the intracellular content with HILIC in the positive and negative ionization mode.

### Pathway analysis

**Fig 3:**
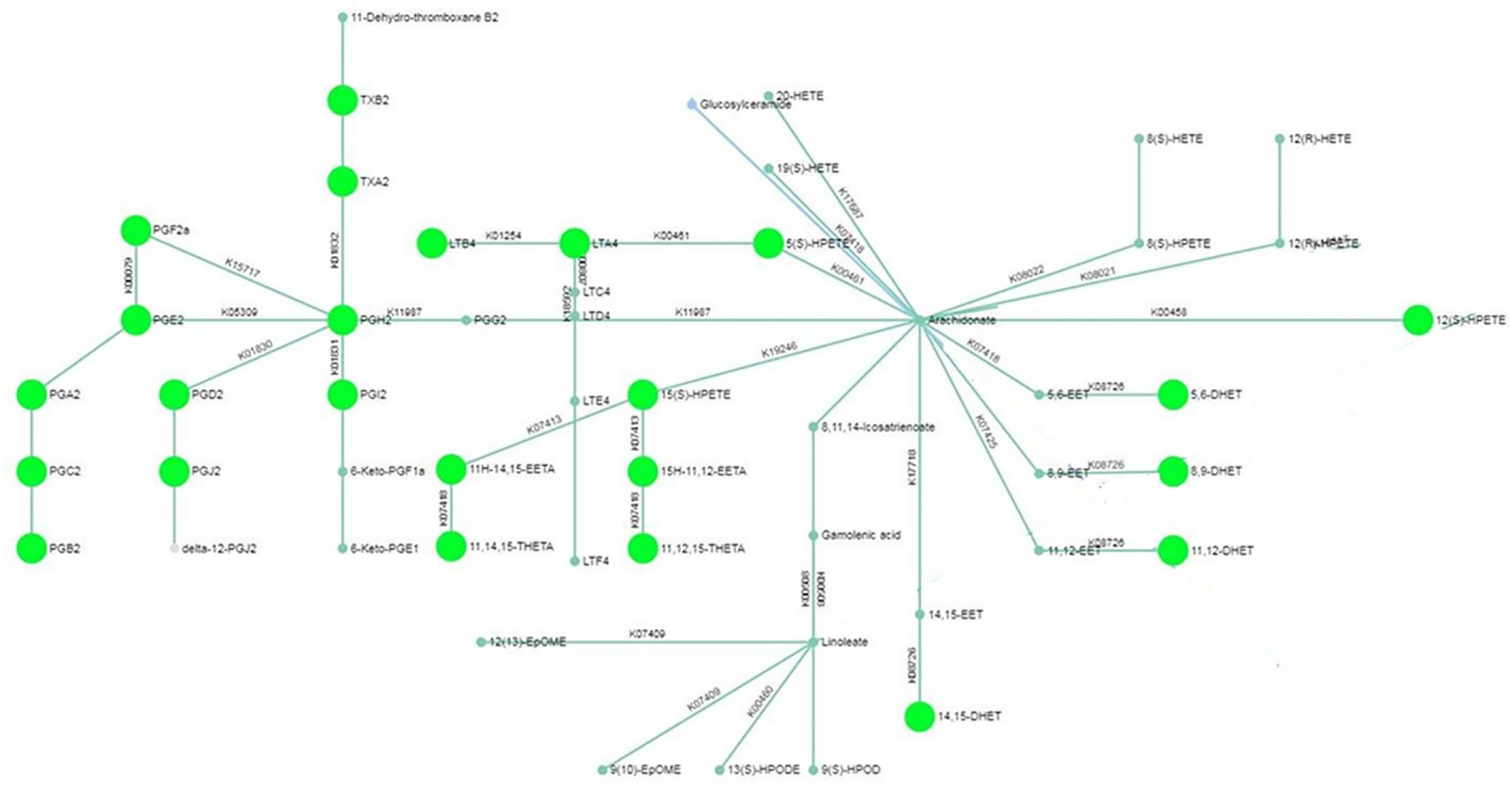
Pathway network mapping for the untargeted metabolomic analysis of intracellular arachidonic acid metabolism in human lung macrophages. Nodes that are significantly enriched in the pathway are fully colored and larger than non-enriched nodes. Pathway analysis was performed with MetaboAnalyst.

Pathway analysis for the LC-MS analysis of the intracellular content revealed differential expression of arachidonic acid metabolites in LPS-treated macrophages (Fig 3). Furthermore, GC-MS analysis suggested modulations in metabolites from tryptophan metabolism and Krebs cycle. Metabolites identified by the statistical analysis to be the most contributing to the different models are shown in Table 3, with most features corresponding to arachidonic acid metabolites. Since the production of arachidonic acid derivatives after stimulation of lung macrophages with LPS is already well documented [38–42], we then focused on the exploration of tryptophan metabolism and Krebs cycle pathway.

**Table 3:** Features differentially expressed between control and LPS samples in cell and supernatant samples.

Feature	*m/z*		RT	Ionization Mode	Method	Log_2_(Fold Change)	Proposed molecular formula	Proposed annotation	Level ID	Δppm
		**Intracellular**								
1	221.0919		18.5	Pos	HILIC	2.3	C_11_H_12_N_2_O_3_	Hydroxy-tryptophan	3	1
2	299.2003		7.3	Pos	HILIC	4.0	C_20_H_26_O_2_	3,4-didehydro-retinoate	3	1
3	301.2163		7.6	Pos	HILIC	5.4	C_20_H_28_O_2_	4-hydroxyretinal	3	0
4	305.0864		14.8	Pos	HILIC	1.2	C_12_H_17_O_9_	-	3	1
5	317.2112		7.9	Pos	HILIC	4.4	C_20_H_28_O_3_	15-deoxy-PGJ2	3	0
6	319.2270		7.7	Pos	HILIC	5.3	C_20_H_30_O_3_	Leukotriene A4	3	1
7	344.2438		18.4	Pos	HILIC	3.7	C_18_H_34_NO_5_		4	2
8	393.2260		7.4	Pos	HILIC	5.7	C_22_H_32_O_6_	10-hydroperoxy-H4-neuroprostane	3	3
9	120.0449		4.9	Neg	HILIC	4.0	C_7_H_7_NO	-	3	4
10	164.0346		4.9	Neg	HILIC	3.8	C_8_H_7_NO_3_	Pyridoxolactone	3	4
11	167.0171		17.7	Neg	HILIC	2.2	C_2_H_5_N_3_O_6_	-	4	7
12	194.0451		3.8	Neg	HILIC	6.7	C_9_H_9_NO_4_	Hydroxyhippuric acid	3	4
13	315.1952		7.2	Neg	HILIC	4.9	C_20_H_28_O_3_	15-deoxy-PGJ2	3	4
14	333.2061		7.1	Neg	HILIC	8.7	C_20_H_30_O_4_	Prostaglandin A2	3	3
15	334.2095		5.2	Neg	HILIC	5.0	C_15_H_30_N_2_O_6_	-	4	4
16	351.2160		7.1	Neg	HILIC	4.8	C_20_H_32_O_5_	Thromboxane A2	3	5
17	353.2325		9.7	Neg	HILIC	2.7	C_20_H_34_O_5_	Prostaglandin E1	3	2
18	368.2432		10.4	Neg	HILIC	5.1	C_18_H_30_NO_5_	-	4	1
19	381.2632		8.9	Neg	HILIC	10.3	C_22_H_32_N_4_O_4_	-	3	4
20	383.2425		5.1	Neg	HILIC	5.5	C_21_H_35_O_6_		4	3
21	385.2217		14.9	Neg	HILIC	2.8	C_20_H_34_O_7_	Dihydro-trihydroxy-leukotriene B4	3	4
22	436.1933		7.0	Neg	HILIC	5.4	C_18_H_26_N_7_O_6_	-	4	3
23	387.1448		18.7	Pos	RP	9.5	C_21_H_22_O_7_	-	3	3
24	134.1		12.40	-	GC	8.4	C_4_H_6_O_5_	Malic acid	1	
25	167.0		15.19	-	GC	1.3	C_7_H_5_NO_4_	Quinolinic acid	1	
		**Extracellular**								
1	136.0398		14.56	Pos	HILIC	8.3	C_7_H_5_NO_2_	-	3	
2	405.2630		7.25	Pos	HILIC	3.4	C_24_H_36_O_5_	-	3	
3	757.4305		7.60	Pos	HILIC	6.3	C_39_H_64_O_14_	-	3	
4	334.2115		6.0	Neg	HILIC	3.9	C_15_H_30_N_2_O_6_		5	2
5	351.2190		6.79	Neg	HILIC	3.6	C_20_H_32_O_5_	Prostaglandin E2	3	

### Targeted metabolic profiling

**Fig 4:**
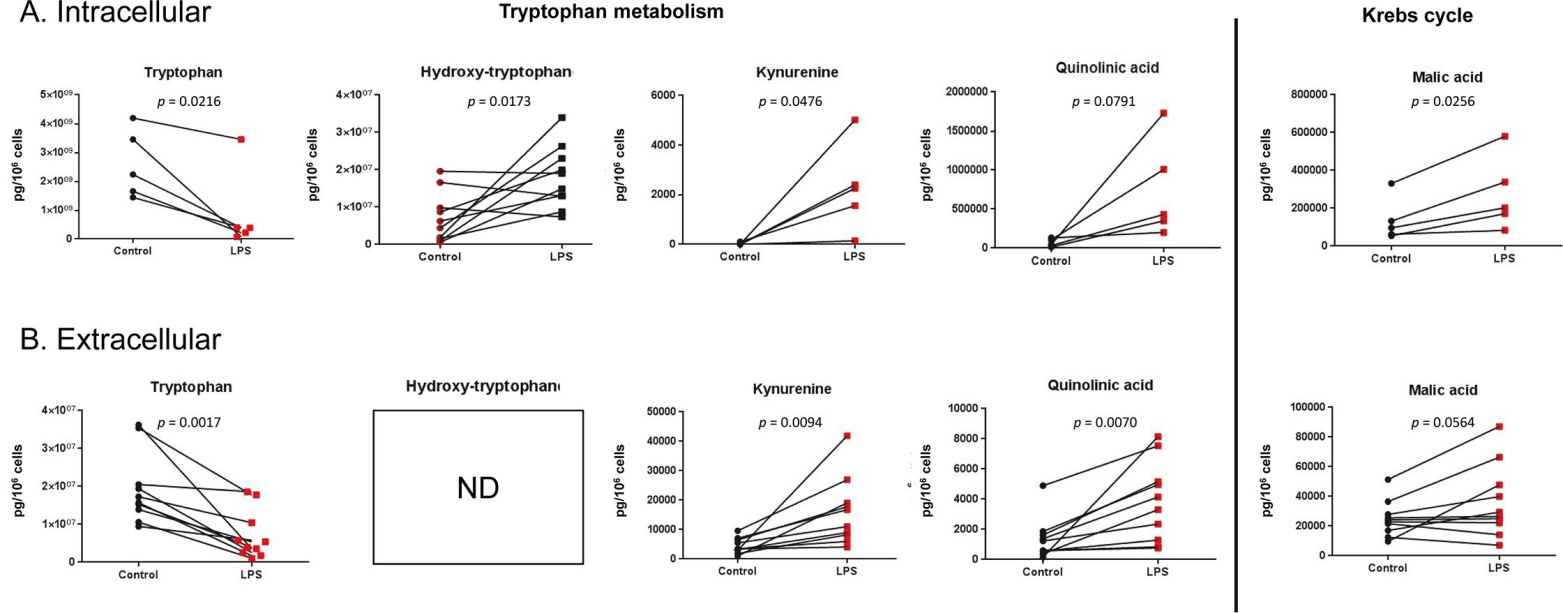
Targeted analysis. Quantification of selected intracellular (A) and extracellular (B) metabolites from tryptophan metabolism and Krebs cycle in human lung macrophages cultured for 24 hrs with or without (control) LPS (10 ng.mL^-1^). Data are expressed in pg per 10^6^ cells. ND: not detected.

Specific targeted LC-MS methods were developed for the quantitative analysis of selected compounds from tryptophan metabolism and Krebs cycle and the results are depicted in Fig 4. For tryptophan metabolism, a LPS-induced decrease in the tryptophan concentration and an increase in concentrations of coumpounds from the kynurenine pathway (kynurenine and quinolinic acid) were observed in both the intracellular and extracellular compartments (quinolinic acid increase was statistically significant in the extracellular content only). Accordingly, the concentration of hydroxytryptophan, a metabolite of the other tryptophan degradation pathway leading to serotonin synthesis, was also increased in the intracellular content. For Krebs cycle metabolites, an increase in the concentration of malate was observed (statistically significant for the intracellular content only), whereas succinate and fumarate were found unaltered. In a similar model with murine bone-marrow derived macrophages [43], the increased malate production was explained by the induction of the arginosuccinate shunt, involving arginosuccinate, fumarate and malate. In line with this, intracellular arginosuccinate was measured in human lung macrophages and a 336% increase in the production was observed (S1 Fig).

## Discussion

This study reports for the first time the metabolomic analysis of human primary lung macrophages, to assess the effect of LPS, the archetypal TLR agonist, on the cell metabolome. The methodological approach combined high-resolution LC-MS, with a split scan-range acquisition method providing improved feature detection when compared to classical methods [23], and GC-MS to provide the most extensive metabolome coverage. Metabolomics studies were previously performed with macrophages to assess the effects of drugs, plasticizers, pollutants and nanomaterials in mouse macrophage cell lines [44–47], to study the interactome in IFN-γ or/and LPS-primed murine macrophages [48] or the cellular metabolism in HIV-infected human monocyte-derived macrophages [49]. Since the availability of human primary lung macrophages is limited and prolonged culture cannot be readily performed, alternative cellular models are commonly used as surrogate to study macrophage biology. Most frequently used surrogates consist in blood monocytes, monocyte-derived macrophages or phorbol ester-differentiated cell lines (e.g. U937, THP-1, HL60). However, important phenotypic differences were already reported between such surrogates and primary macrophages. For example, distinct patterns and expression levels of G-protein coupled receptors (including cytokine receptors) and ion channels were found between monocytes, cell lines and human alveolar macrophages [50]; peripheral blood monocytes exhibit no suppression by LPS of 5-lipoxygenase metabolism and no induction of iNOS compared to alveolar macrophages [41], LPS-stimulated human alveolar macrophages produce more PGE_2_ than do blood monocytes [42] and the LPS-induced cytokine production is also higher in primary lung macrophages than in MDMs [51], highlighting the interest of confirming with primary cells the results obtained with available surrogates.

In addition to molecules from arachidonic acid metabolism, pathway analysis revealed a role for the tryptophan metabolism and Krebs cycle during macrophage M1 polarisation. The role of eicosanoids in the macrophage inflammatory regulation and its resolution is already well established, with increased LPS-induced prostaglandin and leukotriene production [38–40, 52], the inhibitory role of PGE_2_ and regulating role of 15-lipoxygenases in cytokine production [2, 53–55]. Hydroxy-tryptophan, kynurenine and quinolinic acid are downstream metabolites of tryptophan metabolism and were quantified in the present study. Hydroxy-tryptophan is the precursor of serotonin, whereas kynurenine and quinolinic acid belong to the kynurenine pathway. Hence, the depletion of tryptophan is the consequence of both the LPS-induced increase in indoleamine-2,3-dioxygenase (IDO), leading to the formation of kynurenine metabolites, as described in human pulmonary macrophages [56] and to the increase in tryptophan hydroxylase (TPH) activity, also occurring in macrophages in inflammatory conditions [57]. The effectors of the kynurenine pathway, expressed in epithelial cells and alveolar macrophages, were previously shown to be critical regulators of acute pulmonary inflammation in a murine model of lung transplantation [58]. In infectious disease models, IDO expression is increased by respiratory syncytial virus in human monocyte-derived dendritic cells and by IFN-γ and HIV in human monocyte-derived macrophages [59, 60] and quinolinate is increased by HIV in monocyte-derived macrophages [49]. In the clinics, increased IDO and TPH activities were strongly associated to 30-day death and or intensive care unit admission and/or 18 month mortality in patients with COPD exacerbations [61]; an increase in serum kynurenine and a decrease in tryptophan was observed in patients with pneumonia with correlations between IDO activity / kynurenine levels and severity or mortality [62], while kynurenine levels were associated with 28-day mortality in critically ill adult patients [63]. Evidence showing the involvement of tryptophan in the regulation of macrophage polarization is also available. For instance, the role of the kynurenine pathway in inducing changes in macrophage phenotypes was previously investigated in the murine macrophage cell line RAW 264.7 and the murine fibrosarcoma cell line MC57, showing a role for IDO in cell adhesion, metalloproteinase expression and in the expression and activity of the cyclooxygenase enzymes [64]. Culture of RAW264.7 macrophages in a tryptophan-deficient medium induced a 54% reduction in cell proliferation as compared with cells cultured in RPMI, which was restored by tryptophan supplementation. In these cells, tryptophan deficiency was also responsible of an increase in cell death and apoptosis, which was also reversible by tryptophan supplementation [65]. With respect to the production of signaling molecules, macrophages from indoleamine 2,3-dioxygenase 2 knockout mice produced higher amounts of IL-1α, IL-6, IL-10, MCP-1, MIP-1α, MIP-1β and RANTES after LPS stimulation than macrophages from wild-type mice. In line with this, the preincubation of IFN-γ–primed induced pluripotent stem cell–derived human macrophages with INCB024360, an IDO1 inhibitor significantly impaired bacterial killing, which is a key feature of M1-polarized macrophages [66]. RAW264.7 macrophage cells and primary murine alveolar macrophages also showed increased IL-6, TNF-α, IFN-β, and/or IL-1β production in the presence of 1-methyltryptophan another IDO pharmacological competitive inhibitor, following influenza infection [67]. On the other hand, overexpression of IDO enzyme in the murine macrophage cell line RAW264.7 suppressed IL-6, G-CSF, MCP-1, and MIP-1β production [68] while treatment of these cells with the metabolites indole-3-acetate and tryptamine significantly attenuates the expression of TNF-α, IL-1β, and MCP-1 [69]. The production of molecules involved in microbes killing was also affected since LPS and IFN-γ stimulated RAW264.7 cells cultured in tryptophan-deficient medium demonstrated a significant reduction in iNOS expression in comparison to control cells, while cells cultured in the presence of tryptophan expressed significantly higher amounts of iNOS, leading to marked released amounts of NO [65]. Our results also strongly support a major role for tryptophan metabolism in lung macrophages during inflammation, suggesting the interest of a pharmacological approach to modulate this pathway in inflammatory lung diseases.

Then, a large increase in the production of malate, one metabolite from the Krebs cycle, was observed in response to LPS, as previously reported in bone-marrow derived mouse macrophages [43, 48], whereas succinate and fumarate levels were unchanged. Other groups reported either very modest or larger increases in succinate after stimulation with LPS of murine bone-marrow derived macrophages, with key roles for succinate in the induction of IL-1β through HIF-1α [7, 43]. In their M1 polarisation model of murine bone-marrow derived macrophages, Jha and colleagues reported that malate accumulation was related to the induction of the arginosuccinate shunt, a pathway connecting the Krebs cycle with the urea cycle, involving aspartate, arginosuccinate, malate and fumarate. In their model, inhibiting this pathway with aminooxyacetic acid induced a concentration-dependant inhibition of the production of nitric oxide and IL-6, important effectors of antimicrobial activity and inflammatory reaction [43]. In line with these findings, we observed a 336% increase in the production of arginosuccinate in LPS-stimulated human lung macrophages, also suggesting a potential link between metabolism and immune functions in primary human cells.

All the changes in metabolite concentrations measured during the targeted analysis were consistent between the intracellular and extracellular compartments, suggesting that intracellularly produced metabolites may play an autocrine/paracrine role by being released in the cell environment in response to an inflammatory stimuli.

The main limit of the study is related to the patient population and sample size, which was limited to assess the effect of covariates such as age, smoking status or COPD. These covariates are known to affect the response of lung macrophages to TLR agonists; however, these changes greatly vary from one study to another. For example, some studies report increases in the LPS-induced cytokine production of alveolar macrophages from smoking or COPD patients [70, 71] whereas opposite findings were also reported by other groups [72, 73] and in other cases, current smoking status had no effect on the production of cytokines in response to LPS [74, 75]. Altogether, these findings supports the concept whereby the LPS-induced production of cytokines by lung macrophages obtained from COPD, smokers, and healthy adults are similar, although this cannot be directly extrapolated to metabolomic analysis.

In conclusion, we described the use of an extensive combined GC- and LC-MS strategy for the metabolomic profiling of the LPS-induced M1 human lung macrophage polarization. The non-targeted analysis revealed the involvement of the arachidonic pathway, tryptophan metabolism and Krebs cycle during the M1 polarisation. Targeted analysis of selected compounds confirmed these findings and allowed the quantification of the identified metabolites and allowed to precise a role for the aspartate-arginosuccinate shunt. Knowing the role of macrophages in inflammatory lung diseases, a further detailed investigation of alterations occurring in these pathways in cells from patients with asthma or COPD should be of particular interest.

## Supporting information

S1 Table Mass spectrometry parameters for HILIC and reverse phase chromatography with positive or negative electrospray ionisation. (DOCX)

S1 Fig Quantification of intracellular arginosuccinate in human lung macrophages cultured for 24 hrs with or without (control) LPS (10 ng.mL^-1^). Data are expressed in pg per 10^6^ cells.
(TIF)

S1 Data (XLSX)

S2 Data (XLSX)
