# Supplementary material for: Metabolic reprograming of LPS-stimulated human lung macrophages involves tryptophan metabolism and the aspartate-arginosuccinate shunt
Source: PLoS One. 2020 Apr 8;15(4):e0230813. doi: 10.1371/journal.pone.0230813 (PMC7141605; doi:10.1371/journal.pone.0230813)
Supplement: S1 Table — (DOCX) [file pone.0230813.s001.docx]

| **Parameter** | **HILIC +** | **HILIC -** | **RP +** | **RP -** |
| --- | --- | --- | --- | --- |
| Injection time (ms) | 250 | 250 | 100 | 100 |
| Sheath gas flow rate | 60 | 50 | 40 | 50 |
| Auxiliary gas flow rate | 30 | 25 | 25 | 25 |
| Spray voltage (kV) | 2,800 | 2,500 | 4,000 | 2,700 |
| Temperature capillary (°C) | 325 | 275 | 275 | 325 |
| S-lens RF level | 155 | 100 | 100 | 85 |
