# Supplementary figures and images for: Metabolic reprograming of LPS-stimulated human lung macrophages involves tryptophan metabolism and the aspartate-arginosuccinate shunt
Source: PLoS One. 2020 Apr 8;15(4):e0230813. doi: 10.1371/journal.pone.0230813 (PMC7141605; doi:10.1371/journal.pone.0230813)

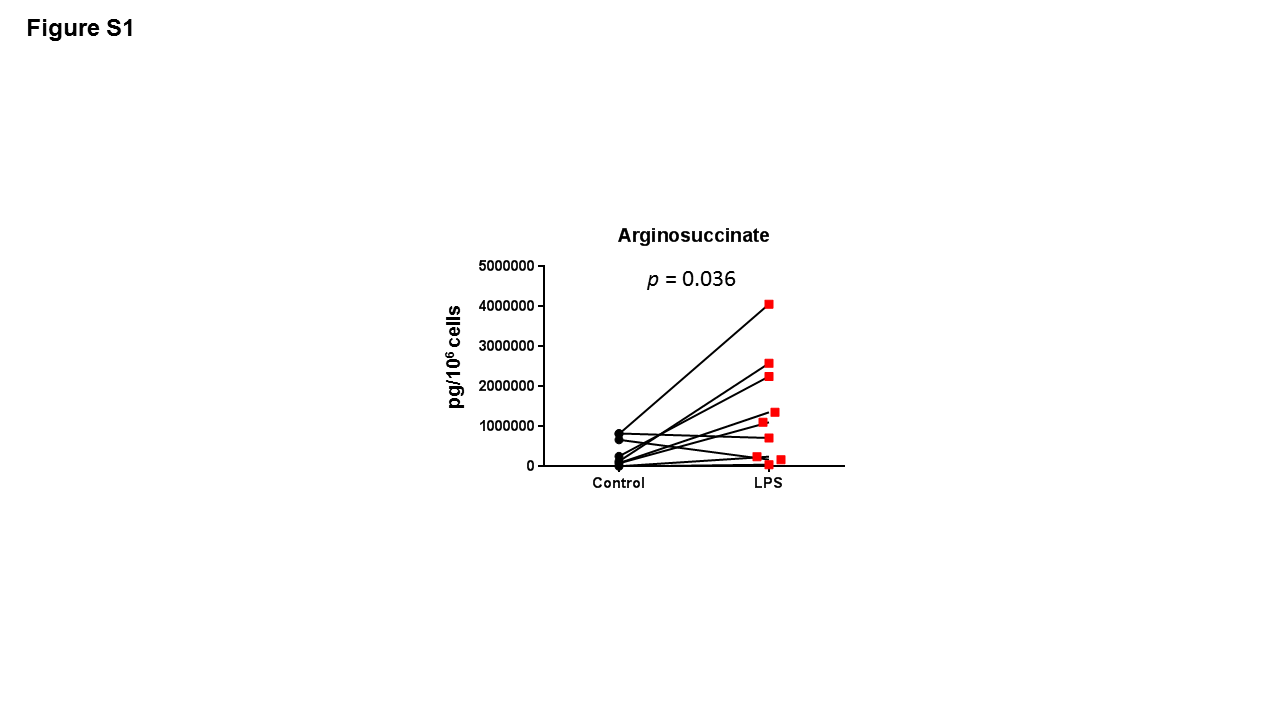

Supplement: S1 Fig — Data are expressed in pg per 106 cells. (TIF) [file pone.0230813.s002.tif]
